# *Lycium barbarum* Glycopeptide prevents the development and progression of acute colitis by regulating the composition and diversity of the gut microbiota in mice

**DOI:** 10.3389/fcimb.2022.921075

**Published:** 2022-08-09

**Authors:** Yichun Huang, Yinghui Zheng, Fengmei Yang, Yicheng Feng, Kunyao Xu, Jun Wu, Shuang Qu, Zhexiong Yu, Fu Fan, Lu Huang, Meng Qin, Zhanlong He, Kaili Nie, Kwok-Fai So

**Affiliations:** ^1^ Beijing Advanced Innovation Centre for Soft Matter Science and Engineering, College of Life Science and Technology, Beijing University of Chemical Technology, Beijing, China; ^2^ Institute of Medical Biology, Chinese Academy of Medical Sciences, Peking Union Medical College, Kunming, China; ^3^ Tianren Goji Biotechnology Co., Ltd, Ningxia, China; ^4^ Guangdong-Hongkong-Macau Institute of Central Nervous System (CNS) Regeneration, Ministry of Education Central Nervous System (CNS) Regeneration Collaborative Joint Laboratory, Jinan University, Guangzhou, China

**Keywords:** ulcerative colitis, gut microbiota, traditional Chinese medicine, 16S rDNA sequence, inflammation

## Abstract

In most cases, recurrent chronic colitis is caused by the recurrence of acute colitis after incomplete recovery and re-exposure to irritating factors, and the gut microbiome, which is the largest micro-ecosystem in the human body, plays a crucial role in the development of colitis. Plant polysaccharides have always been reported to have the ability for anti-inflammation, and they are closely related to the gut microbiome. *Lycium barbarum* Glycopeptide (LbGP), the most potent component obtained by further isolation and purification from *Lycium barbarum* fruit, has been shown to inhibit inflammation in animal models. However, its therapeutic efficacy in colitis and its mechanism in gut microbiota regulation have not been fully studied. In our study, the dextran sulfate sodium (DSS)-induced mouse model was used to dynamically evaluate the effect of LbGP in the treatment of acute colitis and the mechanism from the perspective of the gut microbiome through the 16S rDNA sequence. The results showed that LbGP treatment significantly alleviated acute colitis and improved the gut microbiome compared with that in the model group. Harmful bacteria, such as *Lachnoclostridium* spp. and *Parabacteroides_distasonis*, were inhibited and probiotics, such as *Bacteroides_acidifaciens, Lactobacillus* spp., *Turicibacter* spp., and *Alistipes* spp., were increased by LbGP treatment. Further, a Random Forest analysis with 10-fold cross-validation identified a family named *Muribaculaceae* representing colitis development and recovery upon LbGP treatment. In conclusion, our study demonstrated the capability of LbGP to prevent the development of acute colitis by regulating the composition and diversity of the gut microbiota and highlighted the dynamic process of gut microbiota with the colitis progression. Further, it provides evidence to develop LbGP as a functional food supplement and future drug acting on intestinal disease.

## Introduction

Ulcerative colitis (UC) is a nonspecific inflammatory bowel disease (IBD) characterized by inflammation and ulcer formation in the mucosa and submucosa of the rectum and colon (Mahgoub, 2003). In most cases, colitis is recurrent and it finally becomes a chronic disease that affects the patients for a long period or even for a lifetime (Ordás et al., 2012). The pathogenesis of chronic colitis is currently not known, and it may be related to a variety of biochemical and physicochemical factors. However, in most cases, recurrent chronic colitis is caused by the recurrence of acute colitis after incomplete recovery and re-exposure to irritating factors (Melgar et al., 2005). The gut microbiome plays a crucial role in this progression of the disease. Thus, early intervention and treatment in the acute phase become critical.

The gut microbiome, home to more than 500 species of bacteria, participates in various metabolic pathways and signal transduction in the human physiological processes, and it maintains the stability of the intestinal barrier and the balance of the intestinal environment (Shreiner et al., 2015). In recent years, increasing evidence has supported the claim that gut microbiota plays a critical role in the pathogenesis of colitis (Rooks et al., 2014). Inflammation and damage to the intestinal epithelium caused by exogenous irritants can lead to the influx and colonization of harmful bacteria, which disrupts the balance of the microbiota composition and leads to further disruption of the intestinal barrier function (Janney et al., 2020). Once the intestinal barrier is disrupted, these harmful bacteria can translocate to the lamina propria from the lumen and release toxins and cytokines that promote the development of inflammation (Gallimore and Godkin, 2013).

An increasing number of studies have noticed that plant polysaccharides, such as jujube polysaccharides and *Schisandra chinensis* polysaccharides, not only have an anti-inflammatory effect with few side effects but can also affect the gut microbiome by adjusting the diversity and composition (Ji et al., 2020; Su et al., 2020). It is difficult to directly absorb plant polysaccharides, nondigestible carbohydrates, by the humans gut. Thus, the effects of nondigestible polysaccharides are related to the contribution of the gut microbiota (Xu et al., 2017). Plant polysaccharides can promote the growth of probiotics and restore the balance of the intestinal composition (Xu et al., 2013). They can also be directly or indirectly metabolized by the gut microbiota as nutrients. These metabolites of plant polysaccharides, such as short-chain fatty acids (SCFA), can serve as the main energy source for colon cells, regulate the absorption of various nutrients in the intestine, and enhance the intestinal barrier function (Hu et al., 2018).

The berries of *Lycium barbarum* are generally recognized as natural sources of bioactive compounds, which have nutritional and pharmacological benefits. Dried *Lycium barbarum* fruit is commonly used as a food supplement and traditional Chinese medicine (TCM). It has been found to provide many health benefits, such as antioxidant, anti-inflammatory, and antiapoptotic properties (Manthey et al., 2017). Polysaccharides are the prominent functional ingredients found in *Lycium barbarum* fruit. Modern research has shown that the extract of *Lycium barbarum* fruit has rich medicinal values, and crude polysaccharides extract, *Lycium barbarum* polysaccharide (LBP), is widely used to investigate its biological activities. LBP has been reported to modulate immune function and to be effective in anti-inflammation (Jin et al., 2013; Zhao et al., 2014). It has also been reported to interact with the gut microbiome (Cui et al., 2020; Zhu et al., 2020; Gao et al., 2021). Further separation and purification of natural food supplements and search for active ingredients are needed. *Lycium barbarum* Glycopeptide (LbGP) is an isolated and purified glycoprotein from LBP. It is a kind of glycoconjugate that is considered to be the most potential monomeric component of *Lycium barbarum* fruit. (Peng et al., 2001). Its molecular weight was 88 kDa and its monosaccharide composition contains arabinose (Ara), galactose (Gal), and glucose (Glc) in a molar ratio of 2.5:1.0:1.0. Its protein content was 30% and the linkage between the glycan and the core protein backbone is O-linkage (Tian et al., 1995). The structural characterization was previously analyzed (Wang et al., 2014). Although there were some investigations about the immune activity of LbGP, there was less research into the interaction between LbGP and the intestinal flora. Thus, we decide to investigate the unexplored glycopeptide LbGP’s effects on gut microbiota and its therapeutic effects on UC.

Based on this notion, in this study, acute colitis was induced in mice by 3.5% dextran sulfate sodium (DSS) for one week. We selected a period of 4 weeks to examine the colitis development. We dynamically monitored the progress of physiological indicators and gut microbiome from acute onset to the chronic remission phase, with an aim to determine the function of LbGP in alleviating ulcerative colitis and regulating the gut microbiota.

## Materials and Methods

### Animal subjects

Male C57BL/6J mice weighing around 20 g and aged about 8 weeks were purchased from the Beijing HFK Bioscience Co. Ltd. They were well-nourished and had no history of diseases. All animal care and operations in the experiment followed the regulations of Peking University Institutional Animal Care and Use Committee, and were approved by Animal Ethics Committee of the Institute of Chinese Materia Medica, No. 2021D011.

### Source of *Lycium barbarum* glycopeptide

*Lycium barbarum* glycopeptide (LbGP) was provided by the Ningxia Zhongning Lycium barbarum Academician Workstation. LbGP was accurately extracted from the berries of *Lycium barbarum* through alcohol-free extraction technology, ultra-high-speed sedimentation physical separation technology, and comprehensive membrane separation technology according to the previously described method (Peng et al., 2001). The purity of LbGP was 92.5 ± 3.5% and the HPLC results are shown in **Supplementary Figure S1**.

### Induction of acute colitis with DSS and LbGP treatment

**Figure 1:**
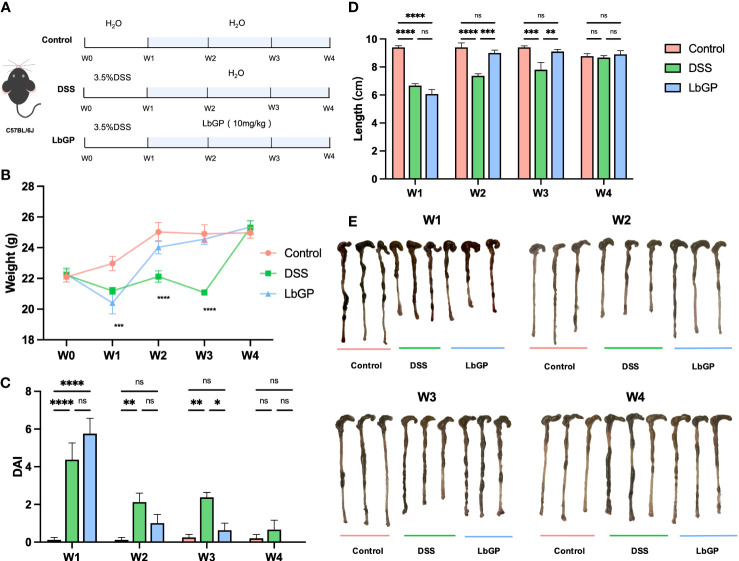
LbGP administration reduced the symptoms of DSS-induced acute colitis in mice. **(A)** Experimental design. **(B)** Bodyweight (n = 6), **(C)** disease activity index (n = 6) and **(D)** length of the colon (n = 3) during the mouse model development. **(E)** Representative colon pictures from each group at different time points. (*P < 0.05, **P < 0.005, ***P < 0.0005, ****P < 0.0001). “ns” means “no significant”.

A total of 45 mice were randomly divided into 3 groups (n=15, each): control group, DSS model group, and LbGP treatment group. As shown in **Figure 1A**, we used four stages to dynamically monitor the therapeutic effect of LbGP on different time periods. The mice were fed with 3.5% DSS dissolved in purified water in the first week (day 0-7) to induce acute colitis. While the control group was fed with normal water. Then, the LbGP treatment group received gavage of LbGP, which was dissolved in sterile saline at a dosage of 10 mg/kg for additional 3 weeks (day 7-28). Meanwhile, the control group was gavaged with sterile saline. At the end of each stage (day 7, day 14, day 21, and day 28, or W1, W2, W3, and W4), the body weights were measured and fecal samples were collected for further experimental analysis. At each time point, three mice that were randomly selected from each group were sacrificed. The blood sample and colon tissues were collected, and the colon length was measured.

### H&E staining and histopathologic analysis

Colon segments measuring 1 cm in length were cut from the aforementioned colon tissues at the upper 2 cm of the anus, washed with PBS solution, and then quickly fixed in 4% paraformaldehyde. The fixed tissues were placed in melted paraffin wax and kept warm, and after the paraffin wax was completely immersed, they were embedded, cooled, solidified, fixed on a microtome, and cut into 5-8 μm thin slices. After removing the paraffin from the sections with xylene, the sections were dehydrated with anhydrous ethanol and rinsed with distilled water, stained with hematoxylin for nuclei and eosin for cytoplasm, and finally sealed with ethanol and xylene for dehydration to complete H&E staining.

### Immunohistochemistry (IHC) staining for inflammatory cell markers

The paraffin sections were deparaffinized, antigens were thermally repaired and, endogenous enzymes were inactivated. Then the sections were blocked and stained with Gr-1 (1:800) and Muc2 (1:500) antibodies (Servicebio, Wuhan, China). The sections were hatched with secondary antibodies, followed by diaminobenzidine (DAB) staining and restaining with hematoxylin. Finally, the stained slides were scanned by Pannoramic SCAN (3DHISTECH Kft, Budapest, Hungary).

### Assessment of the colitis disease activity index (DAI)

The severity of colitis was assessed weekly using a DAI by referring to Cooper et al. (Cooper, 1993). The details of the DAI assessment are shown in **Table 1**. Occult blood in feces was tested using a Urine Fecal Occult Blood Test Kit.

**Table 1:** Assessment of the colitis disease activity index.

Score	Weight loss (%)	Stool consistency	Occult or visible blood in feces
0	0	Normal	Normal
1	1-5	Loose feces	Occult blood positive
2	5-10		
3	10-15	Watery feces	Visible blood
4	>15		

### Measurement of gene expression in colon tissue by quantitative real-time PCR (qRT-PCR)

Mice in all groups were sacrificed, and the colon tissue from the cecum to the anus was obtained. After removing the tissue used for H&E staining, a total RNA extraction kit was used to extract and isolate RNA from the remaining tissues. After the tissue was fully ground, total RNA was extracted and diluted to an appropriate concentration. The Servicebio^®^RT First Strand cDNA Synthesis Kit was used to prepare the reverse transcription system. The reverse transcription program was set as follows: 25°C for 5 minutes, 42°C for 30 minutes, and 85°C for 5 seconds. The 2×SYBR Green qPCR Master Mix (None ROX) was used to perform quantitative fluorescent PCR. The gene sequence was checked from GenBank, and GAPDH was used as the internal reference. The sequences of the forward and reverse primers for GAPDH, IL-1β, and IL-6 are shown in **Supplementary Table S1**.

### Measurement of biochemical indices in colon and serum

The colon tissue was homogenized under ice bath conditions, and the supernatant was taken after centrifugation for further biochemical indices determination. Then malondialdehyde (MDA), superoxide dismutase (SOD), and glutathione peroxidase (GSH-Px) were estimated spectrophotometrically using commercial assay kits (Jiancheng Bioengineering Institute, Nanjing, China) according to the manufacturer’s instructions.

Blood was obtained and serum was taken after centrifugation. The absolute concentration of IL-1β, IL-6, and IL-10 was determined spectrophotometrically using commercial ELISA kits (Sino-UK Institute of Biological Technology, Beijing, China), following the manufacturer’s instructions.

### Fecal DNA extraction and 16S ribosomal DNA gene sequencing

Feces were collected under aseptic conditions on day 7, 14, and 21. Fecal microbial DNA was extracted using the HiPure Soil DNA Kits (Magen, Guangzhou, China) according to the protocols. Afterward, the V3-V4 16S rDNA target region was amplified by PCR using the forward primer 341F 5’-CCTACGGGNGGCWGCAG-3’ and the reverse primer 806R 5’-GGACTACHVGGGTATCTAAT-3’ (the amplicon size was 466), according to a previous study (Guo et al., 2017). Related PCR reagents were obtained from New England Biolabs, USA.

After PCR, the amplicons were extracted and purified using the AxyPrep DNA Gel Extraction Kit (Axygen Biosciences, Union City, CA, U.S.) following the protocols. Finally, paired-end sequencing (PE250) was performed on Illumina Novaseq 6000 according to the standard protocols.

### Data preprocessing and bioinformatic analysis

To obtain high-quality clean reads, quality control was further done to the raw reads using FASTP (Chen et al., 2018), and then paired-end clean reads were merged.

Here, after quality control, we used two separate algorithms for reads clustering. One was the UPARSE algorithm (Edgar, 2013) (version 9.2.64) which clustered the clean reads into operational taxonomic units (OTUs) of ≥ 97% similarity, and the other was the UNOISE3 algorithm (Edgar, 2016) pipeline for denoising (error-correcting) Illumina amplicon reads into amplicon sequence variants (ASVs). The results of our study obtained by the two methods were approximately the same. Thus, in the current study, we chose to use the results of OTU with 97% similarity for the subsequent analysis. UCHIME algorithm (Edgar et al., 2011) was used to remove chimeric tags. Next, the highest abundance tag was selected as the representative sequence within each cluster. Then, the representative OTU sequences or ASV sequences were classified into organisms based on the SILVA database (Elmar et al., 2007) (version 132).

Alpha diversity indexes, such as Chao1, ACE, and Shannon index, were calculated by Usearch (version 10.0.240) and visualized by Graphpad Prism 9 (version 0.1.1). Sequence alignment and phylogenetic trees were constructed using Usearch. Bray-Curtis distances generated by Usearch were used to indicate beta diversity among groups and were visualized in R (version 4.0.5) using the pheatmap package (version 1.0.12). Principal coordinates analysis (PCoA) weighted Bray-Curtis distances was plotted in R using the ape package (version 5.5) and ggplot2 package (version 3.3.5). Between-group Venn plot was performed in Python3.7 using the matplotlib-venn package (version 0.11.6).

The stacked bar plots of the phylum and genus abundance were visualized using the ggplot2 package in the R project, and the species abundance was graphed using Circos (Krzywinski et al., 2009) (version 0.69-3). The differentially expressed OTU was analyzed by the edgeR package (version 3.30.3) in the R project and visualized by ggplot2. Linear discriminant analysis Effect Size (LEfSe) analysis was conducted using LEfSe software (Segata et al., 2011). Random Forest (Liaw and Wiener, 2002) was conducted using the random Forest package (version 4.6.14) and caret package (version 6.0.90) for 10-fold cross-validation in the R project. The KEGG pathway analysis was inferred using PICRUSt (Langille et al., 2013) (version 2.1.4).

### Extraction and measurement of short chain fatty acids (SCFAs)

The standard solutions were prepared by dissolving 100 mg of acetic acid, propionic acid, isobutyric acid, butyric acid, isovaleric acid, and hexanoic acid in 100 mL diethyl ether. Then the standard solution was diluted from 1 mL, 0.75 mL, 0.5 mL, and 0.25 mL to 100 mL respectively to prepare for the standard curve. Fecal samples were added with 2 mL phosphoric acid solution and homogenized by vortex for 2 min. Then the homogenized samples were extracted using 1mL diethyl ether for 10 min and centrifuged at 4000 r/min at 4 ° C for 20min. Repeat the previous step again and the two extracts were mixed together and volatilized to less than 1 mL for the GC-MS analysis.

All assays were performed using the ISQ-LT GC-MS System (Thermo, USA). GC-MS conditions were as follows: the heating program was 100°C (5 min)-5°C/min-150°C (0 min)-30°C/min-240°C (30 min), the flow rate was 1 mL/min, the split ratio was 75:1, the carrier gas was high purity helium, the chromatographic column was TG WAX (30m×0.25mm×0.25μm), the mass spectrometry condition was EI source and the bombardment voltage was 70eV, the temperature of the ion source and injection port was set at 200°C and 240°C, respectively.

### Statistical analysis

Student’s t-test and Welch’s t-test were used to analyze data between two groups. Data with more than two groups at different time points were analyzed using two-way analysis of variance (ANOVA), followed by Tukey’s multiple comparisons test. One-way ANOVA, followed by Tukey’s multiple comparisons test was used to analyze the data with more than two groups. Graphpad Prism 9 (version 0.1.1) and R Studio (version 1.4.1106) were used for statistical analysis. Vegan package (version 2.5.3) in the R project was used to analyze the significance of function between groups.

Data are presented as mean ± SEM. *P* < 0.05 was considered statistically significant.

## Results

### Acute colitis induced by DSS was alleviated by LbGP treatment in the mouse model

As shown in **Figure 1A**, after 3.5% DSS induction for 1 week, mice were treated with 10 mg/kg LbGP. To dynamically evaluate the development of colitis, we measured the physiological and biochemical indicators and collected feces for 16S analysis at the end of each week.

The change in bodyweight is one of the important indicators to evaluate whether the acute colitis model was successfully constructed and whether the treatment was efficient. As shown in **Figure 1B**, the weights in model groups were significantly reduced at W1 because of severe colonic inflammation (*P* < 0.005). Starting from W2, the weights of mice in the LbGP group started recovering and it differed from that in the DSS group. However, even at W3, the weights of mice in the DSS group was still lower than that of mice in the LbGP group (*P* < 0.05). This indicated that LbGP treatment provided early relief from weight loss compared to self-healing.

The main clinical symptoms of UC include persistent diarrhea, hematochezia, and weight loss. Therefore, the DAI was obtained to assess the severity of disease in each group of mice at each stage (**Figure 1C**). At the end of the first week, mice in both the DSS group and the LbGP group developed severe symptoms of colitis (*P* < 0.001). As time progressed, the DAI indices of both DSS and LbGP groups were diminished. But from W2, the DAI index of the LbGP group no longer showed a significant difference from that of the control group (*P* = 0.38); however, the DAI index of the DSS group was still significantly higher (*P* < 0.01), and this significant difference existed until W3. Further, the shortened length of the colon is another important manifestation of acute colitis. Ulcerative inflammation in the colon can cause congestion and edema, resulting in a shortened length of the colon. As shown in **Figure 1E**, the whole colon from the cecum to the anus was taken and straightened. There were significant differences in the length of the colon among the three groups (**Figure 1D**). This was consistent with the results of the pre-mentioned body weight and DAI, which suggested that LbGP can significantly restore the length of the colon in mice with DSS-induced colitis. These results indicated that the LbGP intervention can reduce the symptoms of colitis earlier than self-healing.

**Figure 2:**
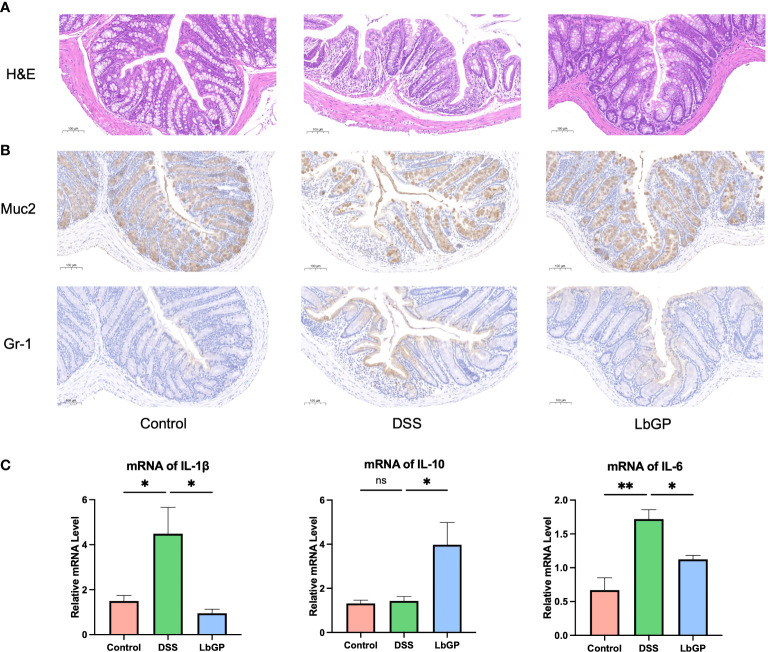
LbGP administration reduced inflammation during DSS-induced acute colitis. **(A)** H&E staining at W2. Scale bar, 100 μm. **(B)** IHC stain of Muc2 and Gr-1 at W2. Representative images are shown. Scale bar, 100 μm. **(C)** Relative mRNA levels of IL-1β, IL-10, and IL-6 at W2 (n=3, *P < 0.05, **P < 0.005). “ns” means “no significant”.

H&E staining demonstrated immune cell infiltration and damage in the colonic tissue. As shown in **Figures 2A, B**, DSS induction led to severe intestinal epithelial damage. Compared with the control group, the DSS group showed intestinal epithelium shedding and ulceration. The mucosal surface was uneven and abnormally distorted, and the crypt fossa morphology was abnormal. The crypt fossa showed distortion or branching, some crypt cyst glands were atrophied. However, after LbGP treatment, the crypt morphology was visible and inflammatory infiltration of the submucosa was less than that in the model group, which proved that LbGP administration obviously reversed the DSS-induced colonic pathological injury.

The colonic mucus layer is critical for maintaining the intestinal barrier and gut homeostasis. Muc2 is a kind of mucin expressed by intestinal epithelial cells, which is an important component of the mucus layer. The Muc2 IHC stain showed that DSS-induced led to a significant decrease of Muc2 and its expression increased after LbGP treatment. Gr-1 is a marker of granulocytes. Its expression can represent the inflammation level and display granulocyte infiltration. The Gr-1 IHC stain showed decreased Gr-1 expression in the LbGP group compared to the DSS group (**Figure 2B**). Those results showed that LbGP restored mucus secretion and alleviate the inflammation.

UC also results in an increase in the pro-inflammatory cytokines. Thus, qRT-PCR was used to measure the cytokine expression levels in colon tissues. As shown in **Figure 2C**, pro-inflammatory cytokines, such as interleukin (IL)-1β and IL-6, could be decreased by LbGP treatment at W2 (*P* < 0.05). Anti-inflammatory cytokines, such as IL-10, were increased after LbGP administration (*P* < 0.05). These results suggested that LbGP had a significant anti-inflammatory effect on DSS-induced colitis. ELISA was used to measure the cytokine levels in serum, which are shown in **Supplementary Figure S2A**. These results were consistent with the PCR results. The cytokine expression levels at W3 were determined to assess the further changes in cytokine mRNA expression (**Supplementary Figure S2B**). It showed that these inflammatory markers turned around quickly. There was no significance in IL-1β, IL-10, and IL-6 between groups, probably because the inflammation induced by acute colitis cannot sustain so long due to self-healing.

Oxidative stress is one of the key factors in UC. Oxidative stress often exhibits a high level of MDA and low levels of SOD and GSH-Px. The levels of MDA, SOD, and GSH-Px in colon tissues at W2 are shown in **Supplementary Figure S2C**. LbGP treatment significantly decreased the MDA level, which indicated its antioxidation effect.

### The gut microenvironment was reestablished by LbGP treatment during DSS-induced acute colitis development

**Figure 3:**
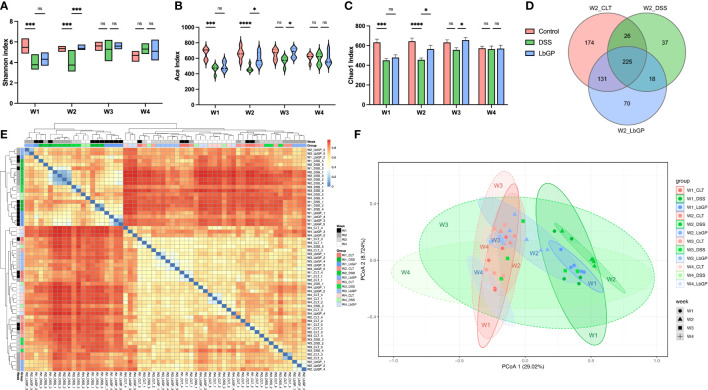
LbGP restored the gut microbial structure in mice with DSS-induced acute colitis. **(A)** Shannon index, **(B)** Ace index, and **(C)** Chao1 index among groups during 3 weeks. **(D)** Venn diagram of the OTU level at W2. **(E)** Heatmap of Bray-Curtis dissimilarity. **(F)** PCoA analysis of Bray-Curtis dissimilarity within PC1 and PC2 axes (n = 5, *P < 0.05, ***P < 0.0005, ****P < 0.0001). “ns” means ”no significant”.

In order to evaluate the dynamic features of the gut microenvironment during LbGP treatment and DSS-induced colitis development, feces were collected at W1, W2, W3, and W4 to perform 16S rDNA sequence. The alpha diversity index indicated the diversity of the gut microbiome within each group. Shannon index, Ace index, and Chao1 index revealed significant reduction in the community richness in the DSS group and LbGP group at W1 because of severe colitis (*P* < 0.005). However, this difference between the Control group and the LbGP group disappeared after LbGP administration at W2 and W3 (**Figures 3A–C**). All the alpha diversity indices in the DSS group also increased over time, but the Ace index and Chao1 index were consistently lower than those in the LbGP group at W3 (*P* < 0.05); thus, showing that LbGP can multiply the gut microbiome and alleviate the imbalance of the gut microbiome faster.

The Venn diagram counted the number of operational taxonomic units (OTUs) with a mean tag count > 1 within each group at W2 (**Figure 3D**). It showed that there were 356 OTUs identical to control in the LbGP group, which was much higher than the 251 OTUs in the DSS group. The Bray-Curtis distance was used to calculate the similarities between samples and was visualized using a heatmap with clustering (**Figure 3E**). The heatmap showed that the DSS group at W1 and W2, and the LbGP group at W1 could be clustered together, which indicated that the model was successfully constructed. Moreover, the LbGP group at W2, W3, W4, and the Control group at all time points could be clustered together, thus, indicating the gut microbiome composition of mice treated with LbGP was closer to that of mice in the Control group.

Principal co-ordinates analysis (PCoA) based on the Bray-Cutis distance demonstrated the same result more intuitively (**Figure 3F**). By observing the distribution of different groups at the same time point on the PCoA plot, it was found that the DSS group had a significant shift along the PC1 compared with the Control group and the LbGP group at W2. However, the location of the LbGP group was close to the Control group. Further, by observing the shift in each group at different time points, it was found that with continuous administration of LbGP, the LbGP group gradually approached the Control group along the PC1. However, the DSS group showed decompaction along the PC1. It showed that there was a large difference within the DSS group at W3 and W4, which may be due to the uncertainty of self-healing (**Supplementary Figures S3A–D**). These results further suggested that LbGP can ameliorate acute colitis by modulating the diversity and structure of the gut microbiota, to promote the repair of murine colonic epithelial injury.

### Altered composition of the gut microbiota by LbGP treatment

**Figure 4:**
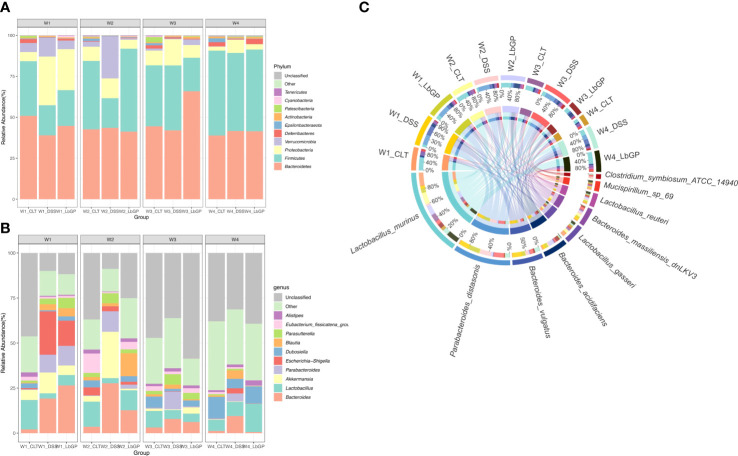
LbGP altered the gut microbiota composition in mice with DSS-induced acute colitis. Relative abundance of the top 10 microbial taxa was determined at the **(A)** phylum level and **(B)** genus level. **(C)** Circos graph of the relative abundance of the top 10 determined species (n = 5).

The dynamic changes in microbiota composition were further investigated. The relative abundance of the top 10 phyla is presented as a stacked histogram (**Figure 4A**). There were notable changes in the relative abundance of *Firmicutes* and *Proteobacteria* in the DSS group and LbGP group compared to the Control group at W1. But at W2, the relative abundance of *Firmicutes* and *Proteobacteria* in the LbGP group returned to normal, while the relative abundance of *Verrucomicrobia* increased shapely in the DSS group, demonstrating that LbGP remarkably boosted *Firmicutes* and inhibited *Proteobacteria* and *Verrucomicrobia*.

To further reveal the genus level changes, the relative abundance of the top 10 genera is presented in **Figure 4B**. DSS treatment induced a significant increase in *Bacteroides*, *Akkermansia, Escherichia-Shigella*, and *Parabacteroides*, while it caused a decrease in *Lactobacillus*. At W2, because of LbGP treatment, the relative abundance of *Bacteroides*, *Escherichia-Shigella*, and *Parabacteroides* decreased, while the relative abundance of *Lactobacillus* increased. At W3 and W4, the genus structure in the LbGP group was nearly identical to that in the Control group. *Escherichia-Shigella* is a common pathogenic bacterium whose early abundance is probably increased due to epithelial damage in the early stages of colitis leading to an influx of pathogenic bacteria. *Parabacteroides* is a kind of bacteria, which is considered to dominate the intestinal bacterial community during the acute phase of colitis (Ezeji et al., 2021). *Lactobacillus* was always considered a probiotic that can produce short-chain fatty acids (SCFAs), such as lactic acid, which is beneficial to the colonic epithelium. Thus, the detailed abundance of *Lactobacillus* and *Parabacteroides* is shown in **Supplementary Figure S4**. The results showed that LbGP administration significantly increased the abundance of *Lactobacillus* and decreased the abundance of *Parabacteroides.*

Concerning the species level, the Circos diagram presented the top 10 identified species (**Figure 4C**). *Lactobacillus_murinus*, *Bacteroides_acidifaciens*, and other *Lactobacillus* spp. were decreased in the DSS-induced groups, and they increased after LbGP administration. However, *Parabacteroides_distasonis* exhibited the opposite phenomenon. *Bacteroides_acidifaciens* is also a kind of probiotic, which has been reported to prevent obesity (Yang et al., 2016). These results were consistent with the aforementioned phylum and genus, suggesting that LbGP treatment can alter the gut microbiota by increasing the abundance of probiotics and decreasing the abundance of harmful bacteria to inhibit the acute development of colitis.

### Identification of signature bacteria in promoting recovery from acute colitis by LbGP treatment

**Figure 5:**
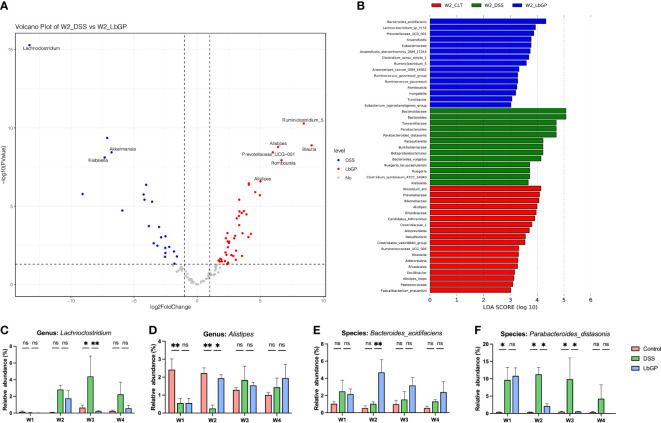
Identification of microbiota biomarker during the development of acute colitis and LbGP treatment. **(A)** Volcano plot of differential OTUs between the DSS group and LbGP group at W2. **(B)** LEfSe analysis among the three groups at W2 with the highest linear discriminant analysis (LDA) score (lg(LDA score) ≥ 3). **(C, D)** Relative abundance of Lachnoclostridium and Alistipes. **(E, F)** Relative abundance of Bacteroides_acidifaciens and Parabacteroides_distasonis (n = 5, *P < 0.05, **P < 0.005). “ns” means “no significant”.

To further identify the key microbiota during LbGP treatment and DSS-induced colitis development, we used moderated statistical tests (Robinson and Smyth, 2007) to assess the tag abundance of OTUs between the DSS group and LbGP group at W2. A volcano plot was presented with a threshold of *P* < 0.05 and |log_2_FoldChange| > 1 (**Figure 5A**). With a cut-off value of minimal tag counts > 10 and minimal total counts > 15, 44 OTUs were enriched in the LbGP group and 23 OTUs were enriched in the DSS group. The identified genera with the top 10 fold-changes were labeled on the plot and the details are shown in **Table 2**. *Clostridium_symbiosum_ATCC_14940*, enriched in the DSS group at W2, belongs to *Lachnoclostridium*, which was recently reported as a biomarker for the diagnosis of colorectal adenoma and cancer (Li et al., 2020). The relative abundance of *Lachnoclostridium* is shown in **Figure 5C**. Interestingly, *Lachnoclostridium* did not colonize the intestine from the onset of colitis, but its abundance increased with the development of acute colitis, and LbGP treatment could prevent its colonization. *Klebsiella* sp. and *Akkermansia* sp. were also enriched in the DSS group. *Klebsiella* was reported to induce gut inflammation (Atarashi et al., 2017). *Akkermansia* spp. was enriched in the DSS group at W2. It was always regarded as a kind of probiotic in obesity and diabetes. However, some previous research also reported that it is increased in DSS-treated mice (Ménard et al., 2004; Zhang et al., 2016). *Alistipes*, *Ruminiclostridium_5*, *Blautia*, *Prevotellaceae_UCG_001*, and *Romboutsia* were enriched in the LbGP group. The relative abundance of *Alistipes* is shown in **Figure 5D**. LbGP administration could restore the relative abundance levels of *Alistipes* (*P* < 0.005). *Blautia* showed potential probiotic properties by preventing inflammation and promoting SCFAs production and other activities to maintain intestinal homeostasis (Liu et al., 2021). These microbiotas could play a significant role during LbGP treatment and were remarkably influenced by LbGP administration.

**Table 2:** Details of differential OTUs between DSS and LbGP group at W2.

OTU	logFC	*P*	FDR	level	Phylum	Family	Genus	Species
OTU000066	-13.29	5.23E-16	6.27E-14	DSS	*Firmicutes*	*Lachnospiraceae*	*Lachnoclostridium*	*Clostridium_symbiosum_ATCC_14940*
OTU000108	8.45	5.21E-11	3.13E-09	LbGP	*Firmicutes*	*Ruminococcaceae*	*Ruminiclostridium_5*	
OTU000267	-7.13	4.37E-10	1.75E-08	DSS				
OTU000059	9.08	1.31E-09	3.93E-08	LbGP	*Firmicutes*	*Lachnospiraceae*	*Blautia*	
OTU000130	6.42	1.65E-09	3.96E-08	LbGP	*Bacteroidetes*	*Rikenellaceae*	*Alistipes*	*Alistipes_inops*
OTU000041	6.00	3.62E-09	6.23E-08	LbGP	*Bacteroidetes*	*Prevotellaceae*	*Prevotellaceae_UCG-001*	
OTU000002	-6.78	3.64E-09	6.23E-08	DSS	*Verrucomicrobia*	*Akkermansiaceae*	*Akkermansia*	
OTU000049	-7.32	7.67E-09	1.15E-07	DSS	*Proteobacteria*	*Enterobacteriaceae*	*Klebsiella*	
OTU000047	6.69	1.17E-08	1.56E-07	LbGP	*Firmicutes*	*Peptostreptococcaceae*	*Romboutsia*	
OTU000101	5.02	2.63E-07	3.16E-06	LbGP	*Bacteroidetes*	*Rikenellaceae*	*Alistipes*	

Linear Discriminant Analysis (LDA) effect size is a method to identify signature biomarkers. **Figure 5B** shows the results of LEfSe analysis among the Control group, DSS group, and LbGP group at W2 with the lg(LDA score) ≥ 3, and its cladogram is presented in **Supplementary Figure S5**. *Bacteroides_acidifaciens*, *Lachnoclostridium_sp_YL32*, *Prevotellacese_UCG_001*, *Anaerofustis*, *Ruminiclostridium_5, Romboutsia*, and *Turicibacter* were enriched in the LbGP group. However, *Parabacteroides_distasonis*, *Burkholderiaceae*, *Klebsiella*, *Parasutterella*, and *Clostridium_symbiosum_ATCC_14940* from *Lachnoclostridium* were enriched in the DSS group. Probiotics were enriched in the Control group. These results roughly comprised the previous volcano plot, which suggested that probiotics were increased in the LbGP group and harmful microbiota was inhibited by LbGP treatment. The two species, *Bacteroides_acidifaciens* and *Parabacteroides_distasonis*, discussed previously, were found again. **Figures 5E, F** show the changes in their relative abundance at different time points among the 3 groups. LbGP treatment significantly increased the abundance of *Bacteroides_acidifaciens* and inhibited the colonization of *Parabacteroides_distasonis*. In this way, we identified several signature bacteria that were associated with LbGP treatment.

### *Muribaculaceae* may act as the microbiota marker for LbGP treatment and colitis development

Apart from the statistical method and LDA analysis, in the cause of finding the implicitly different microbiota markers between groups during the LbGP treatment, a Random Forest model was established. In order to increase the amount of training data for the algorithm to have a good performance, the groups need to have intra-group consistency and to be representative of the gut microbiota in the Control group, the DSS-induced group, and the LbGP-treated group. Considering that there were individual differences in the process of self-healing. Thus, the samples that are close to other groups or the samples that have large differences within the group need to be excluded from this algorithm. After removing samples at W4 and the DSS group samples at W3, due to the uncertainty of the self-healing process in the DSS group, 40 samples remained behind. These 40 samples were divided into the following three categories as training samples for supervised learning: CTL (all of the Control groups, represented the normal state of gut microbiota, n=15), DSS (two model groups at W1 and the DSS group at W2, represented the colitis state of gut microbiota, n=15), and LbGP (the LbGP groups at W2 and W3, represented the treatment state of gut microbiota, n=10).

**Figure 6:**
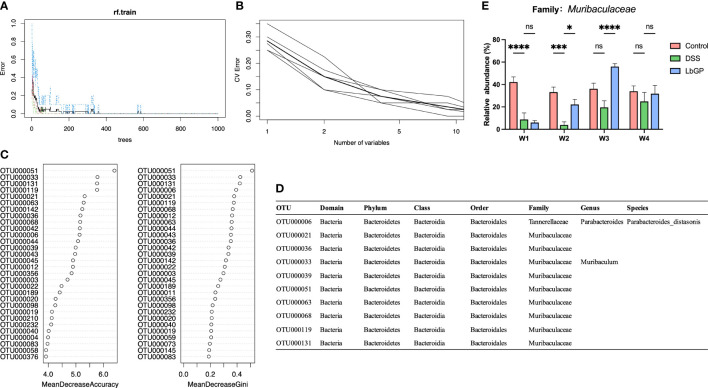
Identification of key OTU using Random Forest classification. **(A)** Error rates of the Random Forest model. **(B)** Error rates of 10-fold cross validation with 5 trials. **(C)** The mean decrease accuracy and mean decrease Gini of the top 20 OTUs in the Random Forest model. **(D)** Detailed taxonomy of the top 10 OTUs in the Random Forest model. **(E)** Relative abundance of Muribaculaceae (n = 5, *P < 0.05, ***P < 0.0005, ****P < 0.0001). “ns” means “no significant”.

As shown in **Figure 6A**, with an increase in the number of trees, the out-of-bag (OOB) error decreased rapidly and approached 0, which indicated that the model showed a good performance. The 10-fold cross-validation with 5 trials (**Figure 6B**) revealed that 10 variables were enough to ensure accurate model prediction. Thus, considering the OTUs with the highest Mean Decrease Accuracy and Mean Decrease Gini (**Figure 6C**), 10 optimal OTUs were identified (**Figure 6D**). Except for the OTU000006 (Parabacteroides_distasonis) which was previously mentioned, all the other OTUs belong to a family named *Muribaculaceae*. *Muribaculaceae* is a newly named family in recent years, previously known as *S24-7* (Lagkouvardos et al., 2019). The detailed abundance of *Muribaculaceae* is shown in **Figure 6E**. The abundance of *Muribaculaceae* increased with LbGP treatment and decreased with DSS-induced colitis development. This result suggested that *Muribaculaceae* can be used as a flora marker for LbGP treatment and the development of colitis.

### Altered metabolism of the gut microbiota during LbGP treatment

**Figure 7:**
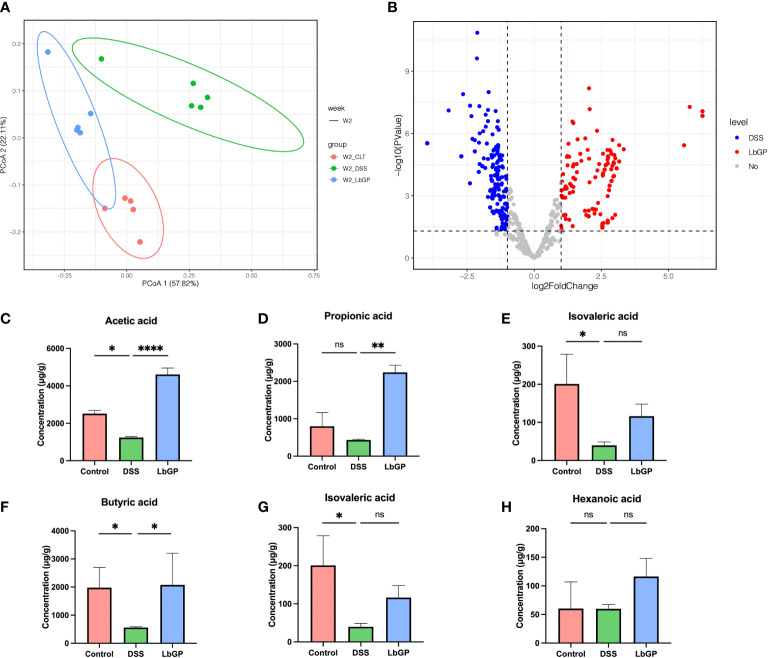
LbGP altered the function of gut microbiota. **(A)** PCoA analysis of metagenomic function based on the Bray-Curtis distance at W2. **(B)** Volcano plot of differential KOs between the DSS group and LbGP group at W2. **(C–H)** Concentrations of different SCFAs in mice colonic contents at W2. (n = 5, *P < 0.05, **P < 0.005, ****P < 0.0001). “ns” means “no significant”.

The PICRUSt algorithm was used to assess the functional difference in bacteria among different groups, and the Kyoto Encyclopedia of Genes and Genomes (KEGG) database was used to describe every possible pathway. Using the Bray-Cutis distance-based PCoA analysis at W2 among the 3 groups (**Figure 7A**), we found that the DSS group was significantly shifted along both PC1 and PC2 compared with the Control group, while the LbGP group only shifted along PC2. It showed that the metagenomic function of the gut microbiota in the LbGP group was similar to that in the Control group.

Then a statistical method was used to analyze the different types of KEGG Orthology (KO) between the LbGP group and DSS group at W2. With a threshold of *P* < 0.05 and |log_2_FoldChange| > 1, there were 412 KOs enriched in the LbGP group and 407 KOs enriched in the DSS group (**Supplement Figure 7B**), which showed that LbGP treatment significantly altered the metabolism compared to the model. Further, Welch’s T-test was used to estimate the different KEGG metabolic pathways in level 3 with *P* < 0.01 (**Supplementary Figures S6A, B**). The result showed that DSS induction significantly increased most of the pathways, which disrupted the homeostasis of the gut microbiota function. The overall upregulation of these functional pathways may be due to the functional compensation due to inflammation. After LbGP administration, the increase reversed, which showed that LbGP treatment may restore the balance of metagenomic function by re-establishing the microenvironment.

In addition to the functional prediction of gut microbiota, SCFAs analysis was carried out to study the effect of LbGP on the microbial metabolic product. The concentrations of acetic acid, propionic acid, isobutyric acid, butyric acid, isovaleric acid, and valeric acid in mice colonic contents at W2 are shown in **Figures 7C–H**. After the DSS-induce, the content of acetic acid, butyric acid, and isovaleric acid significantly decreased in the DSS group mice compared to the Control group (*P* < 0.05). SCFAs level recovered after one-week LbGP treatment. Especially the content of acetic acid, propionic acid, and butyric acid was significantly higher in the LbGP group mice than the DSS group mice (*P* < 0.05).

## Discussion

The development of colitis is a dynamic and continuous process, and timely treatment is very important for recovery in the acute phase of colitis. In most cases, acute colitis has a tendency to heal spontaneously; however, the intestinal epithelial damage and dysregulation of the internal environment during the acute phase persist in the intestine (Sartor, 2006). They are more likely to recur the next time when it is stimulated by environmental factors. Here, we used a long-term observation to assess the development of LbGP treatment and colitis itself in order to determine their effects on the diversity, composition, and some signature bacteria of the intestinal flora in DSS-induced colitis.

This long-term observation allowed us to understand the dynamic pathological process from acute to chronic remission of UC, especially the process of early colonization and late abundance changes in the gut microbiota. As discussed earlier, although the alpha diversity and composition of the DSS model group were getting better in the last week, the beta diversity showed that the recovery was still unstable. This may lead to incomplete recovery of the intestinal environment and poor protection against the next stimulus. Further, being a harmful bacterium, the colonization of *Lachnoclostridium* sp., as mentioned earlier, did not occur at the beginning of colitis. But it gradually colonized the intestine as colitis progressed and its abundance gradually increased. Even at W3, when the other indicators seemed to have recovered, the abundance of *Lachnoclostridium* was still increased in the model group. Thus, this may suggest that this bacterium may be associated with the ease of recurrence of colitis. These results demonstrate that self-restoration is inadequate for intestinal microbiome disorders caused by acute colitis, which may be one of the reasons why UC is vulnerable to recurrence.

In the current study, LbGP treatment could remarkably re-establish the bacterial species and quantity, and finally, restore the homeostasis in the intestine, which may prevent the recurrence of colitis. Thus, we next identified some of the signature bacteria during LbGP treatment and colitis development. In the current study, we found that LbGP treatment could significantly increase the abundance of probiotics, such as *Bacteroides_acidifaciens, Lactobacillus* spp., *Turicibacter*, and *Alistipes*, and decrease the abundance of harmful bacteria, such as *Lachnoclostridium* spp. and *Parabacteroides_distasonis*. These probiotics were reported to produce SCFAs and promote repair of the intestinal mucosal barrier, which accelerated colonic repair (Kles and Chang, 2006). In our founding, the concentration of SCFAs suddenly decreased during UC, while LbGP treatment can significantly improve most of the SCFAs. It proves the improvement of probiotics leads to an increase in SCFAs. SCFAs can bind to the G-protein-coupled receptor and exhibit an anti-inflammatory effect (Maslowski et al., 2009). For example, acetic acid was reported as an energy source for intestinal cells and it can regulate the inflammatory response and maintain the mucosal barrier (Ménard et al., 2004). Propionic acid and butyric acid were reported to have the ability to maintain the stability of intestinal microecology (Vinolo et al., 2011), improve intestinal immunity, and maintain intestinal homeostasis (Furusawa et al., 2013). This suggests that SCFA can be the bridge between gut microbiota and colitis recovery during LbGP treatment.

Most interestingly, by applying a machine-learning method, we found several species from a family recently named *Muribaculaceae*, which can together distinguish the non-treatment control group, DSS-induced colitis group, and LbGP administration group. It is worth noting that the Random Forest analysis did not indicate the effect of a species from *Muribaculaceae*. Still, it indicated the overall result of some species in this family. A recent study reported that *Muribaculaceae* was increased during Ginseng polysaccharide treatment, which indicates that plant polysaccharides may affect *Muribaculaceae* (Huang et al., 2022). It was difficult to isolate and culture the species in *Muribaculaceae in vitro*, and there were few annotations of this family in the database. It is difficult to detect the relationship between this flora and disease by traditional statistical methods, but machine learning can serve this purpose. In our study, we found that *Muribaculaceae* was remarkably reduced in mice with DSS-induced colitis and was restored by LbGP treatment; this proves that *Muribaculaceae* played a significant role in colitis development and its recovery.

As mentioned before, LbGP is obtained by further purification of LBP. Its effect on UC and gut microbiota is consistent with that of LBP to some extent. This suggested that glycopeptide acts as the primary component of LBP in colitis treatment. Similar to LBP (Zhao et al., 2014; Zhu et al., 2020), LbGP can reduce inflammation and improve the diversity of gut microbiota. Both of them can increase some probiotics, such as *Lactobacillus* and *Prevotellaceae*. However, there are still some differences between LbGP in our study and LBP. For example, *Akkermansia* was downregulated by LbGP, but it was increased by LBP (Zhu et al., 2020). *Alistipes* was increased in our study, but it was downregulated by LBP (Cui et al., 2020). On the one hand, these differences may be caused by the differences in disease models. Because different disease models lead to different compositions of the gut microbiota, which in turn affects the status and function of different bacteria. On the other hand, LBP extracted by the conventional process yields far less macromolecular contents and far less protein, while LbGP has more glycoprotein structure which means higher bioactivity. These glycoproteins were hardly digested by the human gut but can be degraded by some specific bacteria (Rhodes et al., 1985). These glycoprotein metabolites, such as oligopeptides and amino acids, can serve as a major nutrient source for some colonic bacteria, thus altering the microbiota composition (David Sela and Underwood, 2012). Some bacteria, such as *Alistipes*, can use amino acids for fermentation and produce a more diverse range of metabolites, including SCFAs (Yao et al., 2016), which influence the intestinal environment. Therefore, these differences need to be observed in further treatment and investigation.

Furthermore, our findings are highly consistent in all aspects and will help to test the future clinical use of this bioactive food ingredient for treating gastrointestinal disease. The alpha diversity results were highly consistent with the DAI index and colon length. This suggested that the destruction and recovery of intestinal microbiota were consistent with the entire pathological process during colitis development and LbGP treatment. This provided evidence for the synergy between the intestinal microbiota and disease phenotypes.

## Conclusions

In conclusion, our results indicated that LbGP can serve as a potential natural extract of TCM to treat UC and prevent the transformation from the acute to the chronic phase. In future studies, metabonomics and metagenomics can be combined to dig deeper into the metabolic patterns of the gut microbiota under the influence of LbGP treatment. Fecal transplants can be further used to explore the therapeutic effects of probiotics discussed in this study. In particular, future investigation to determine the detailed mechanistic aspect of the relationship between *Muribaculaceae* and colitis will be meaningful.

## Code availability

Code for bioinformation process and plotting of this paper was available at https://github.com/YCHuang0610/LbGP-gut-microbiome

## Data availability statement

The datasets presented in this study can be found in online repositories. The names of the repository/repositories and accession number(s) can be found below: https://ngdc.cncb.ac.cn/gsa, CRA005565.

## Ethics statement

The animal study was reviewed and approved by Animal Ethics Committee of the Institute of Chinese Materia Medica.

## Author contributions

Author contributions: YH, and YZ carried out most experiments under the guidance of ZH and KN. FF, ZY, and JW assisted with the extraction and purification of the Lycium barbarum Glycopeptide. YF, KX, FY, MQ, and SQ helped to accomplish the animal experiments. YH was responsible for the bioinformatic analysis. K-FS and LH carried out article correction and provide guidance on the overall study. All authors discussed the results and commented on the manuscript. All authors contributed to the article and approved the submitted version.

## Conflict of interest

Authors ZY and FF were employed by company Tianren Goji Biotechnology Co., Ltd.

The remaining authors declare that the research was conducted in the absence of any commercial or financial relationships that could be construed as a potential conflict of interest.

## Publisher’s note

All claims expressed in this article are solely those of the authors and do not necessarily represent those of their affiliated organizations, or those of the publisher, the editors and the reviewers. Any product that may be evaluated in this article, or claim that may be made by its manufacturer, is not guaranteed or endorsed by the publisher.
