# Corrigendum: *Lycium barbarum* Glycopeptide prevents the development and progression of acute colitis by regulating the composition and diversity of the gut microbiota in mice

**DOI:** 10.3389/fcimb.2022.1021676

**Published:** 2022-11-17

**Authors:** Yichun Huang, Yinghui Zheng, Fengmei Yang, Yicheng Feng, Kunyao Xu, Jun Wu, Shuang Qu, Zhexiong Yu, Fu Fan, Lu Huang, Meng Qin, Zhanlong He, Kaili Nie, Kwok-Fai So

**Affiliations:** ^1^ Beijing Advanced Innovation Centre for Soft Matter Science and Engineering, College of Life Science and Technology, Beijing University of Chemical Technology, Beijing, China; ^2^ Institute of Medical Biology, Chinese Academy of Medical Sciences, Peking Union Medical College, Kunming, China; ^3^ Tianren Goji Biotechnology Co., Ltd, Ningxia, China; ^4^ Guangdong-Hongkong-Macau Institute of Central Nervous System (CNS) Regeneration, Ministry of Education Central Nervous System (CNS) Regeneration Collaborative Joint Laboratory, Jinan University, Guangzhou, China

**Keywords:** ulcerative colitis, gut microbiota, traditional Chinese medicine, 16S rDNA sequence, inflammation

## Error in figure/table

In the published article, there was an error in **Figure 7** as published. **Figure 7E** was incorrectly placed as **Figure 7G** and **Figure 7G** appeared twice by mistake. The corrected **Figure 7** and its caption appear below.

**Figure 7:**
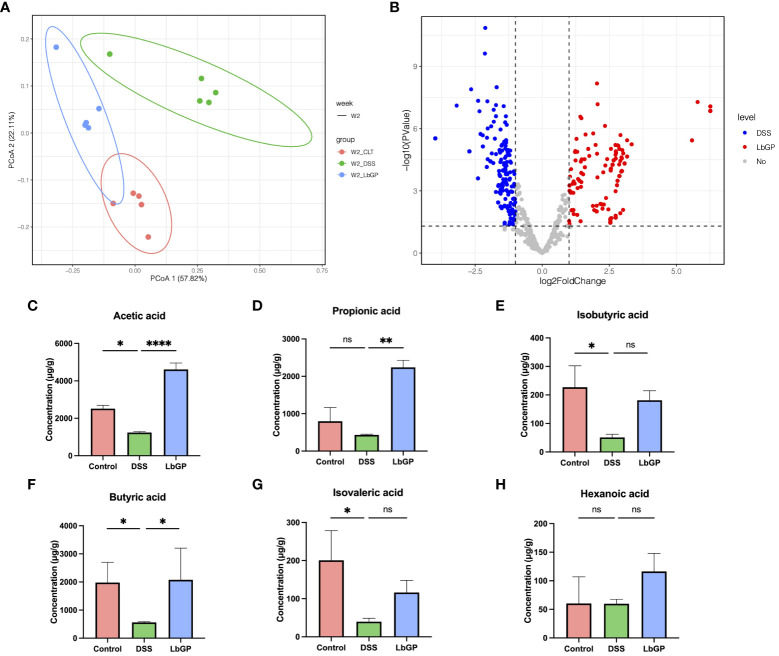
LbGP altered the function of gut microbiota. **(A)** PCoA analysis of metagenomic function based on the Bray-Curtis distance at W2. **(B)** Volcano plot of differential KOs between the DSS group and LbGP group at W2. **(C–H)** Concentrations of different SCFAs in mice colonic contents at W2. (n = 5, *P < 0.05, **P < 0.005, ****P < 0.0001). “ns” means “no significant”.

The authors apologize for this error and state that this does not change the scientific conclusions of the article in any way.

## Publisher’s note

All claims expressed in this article are solely those of the authors and do not necessarily represent those of their affiliated organizations, or those of the publisher, the editors and the reviewers. Any product that may be evaluated in this article, or claim that may be made by its manufacturer, is not guaranteed or endorsed by the publisher.

