# Supplementary material for: Lycium barbarum Glycopeptide prevents the development and progression of acute colitis by regulating the composition and diversity of the gut microbiota in mice
Source: Front Cell Infect Microbiol. 2022 Aug 9;12:921075. doi: 10.3389/fcimb.2022.921075 (PMC9395742; doi:10.3389/fcimb.2022.921075)
Supplement: Supplementary file 1 [file DataSheet_1.docx]

Supplementary Materials

# Supplement Method

## High-performance liquid chromatography analysis of LbGP

The ultrapure water and 50mM Na_2_HPO_4_ solution were filtered through a 0.45um aqueous filter membrane before use. 20μL Na_2_HPO_4_ solution was used as blank control. 5 g LbGP was dissolved with 1 mL of 50 mM Na2HPO.

The chromatographic conditions were as follows: the chromatographic column was TSK gel G3000 PWXL, the mobile phase was 50 mM Na2HPO4, the pH was 6.8, the flow rate was 0.5 ml/min, the column temperature was 30 °C, the detection wavelength was 260 nm, the column pressure was 1.5 MP, and the detection time was 30 min.

# Supplementary Figures


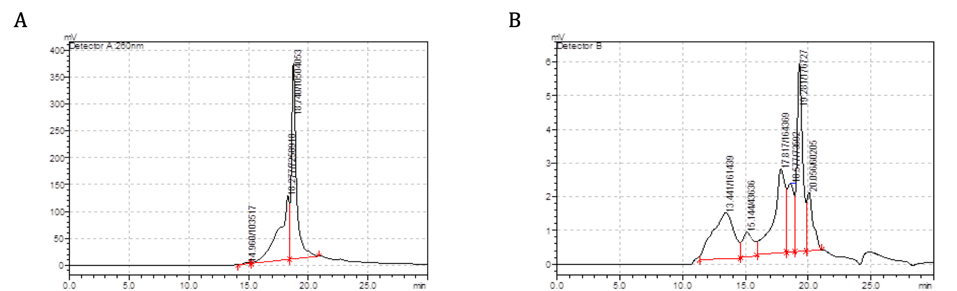


**Supplementary Figure S1.** HPLC analysis of LbGP. (A) Specific chromatogram at UV 260nm. (B) Infrared-specific chromatogram.


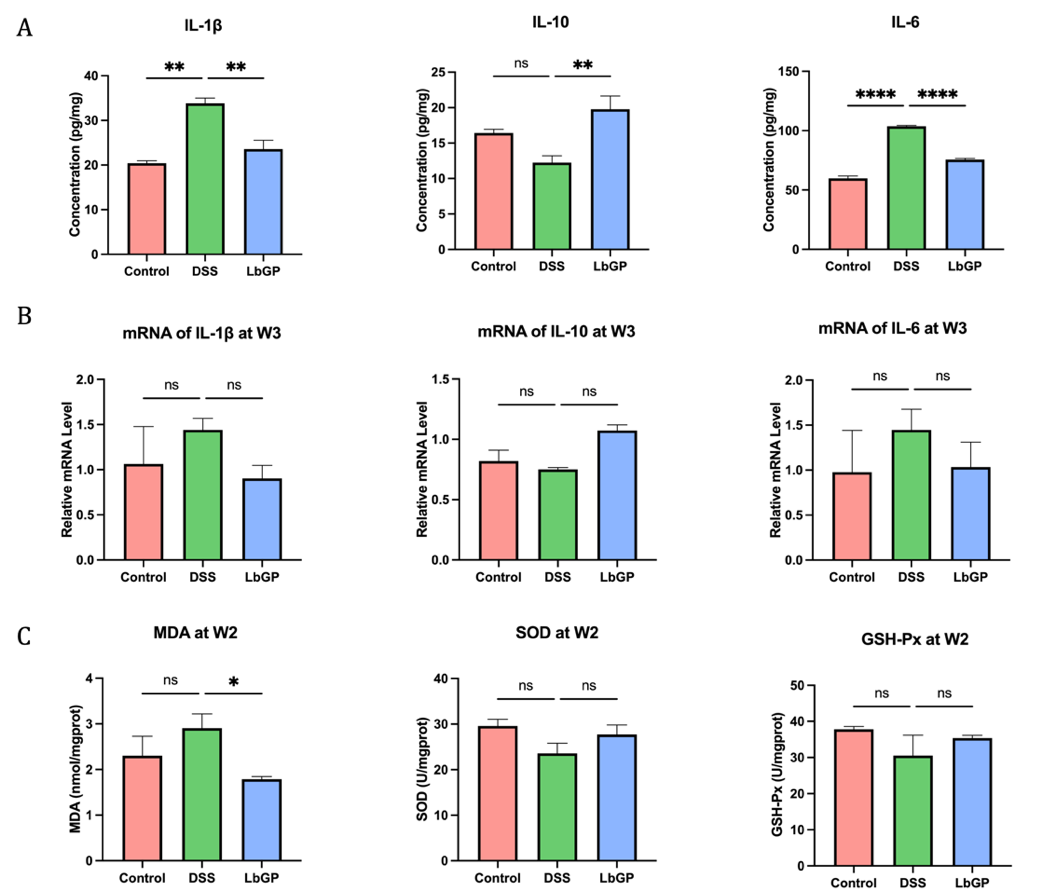


**Supplementary Figure S2.** Cytokine levels and biochemical indices in serum and colon tissues. (A) Cytokines level in serum at W2. (B) The relative mRNA expression level of cytokines at W3. (C) Biochemical indices in colon tissues at W2. (n=3, **P*<0.05, ***P*<0.005)


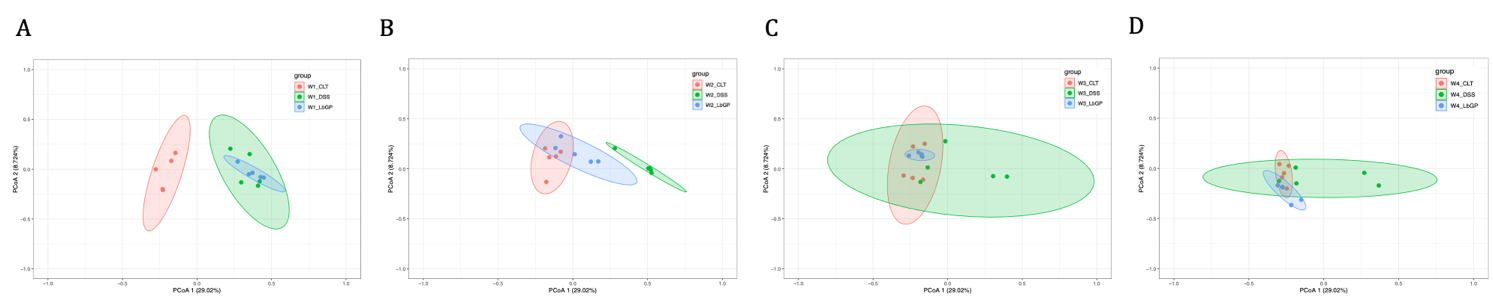


**Supplementary Figure S3.** PCoA analysis of Bray-Curtis dissimilarity within PC1 and PC2 axes among different groups at (A) W1, (B) W2, (C) W3, and (D) W4 (n=5).

#
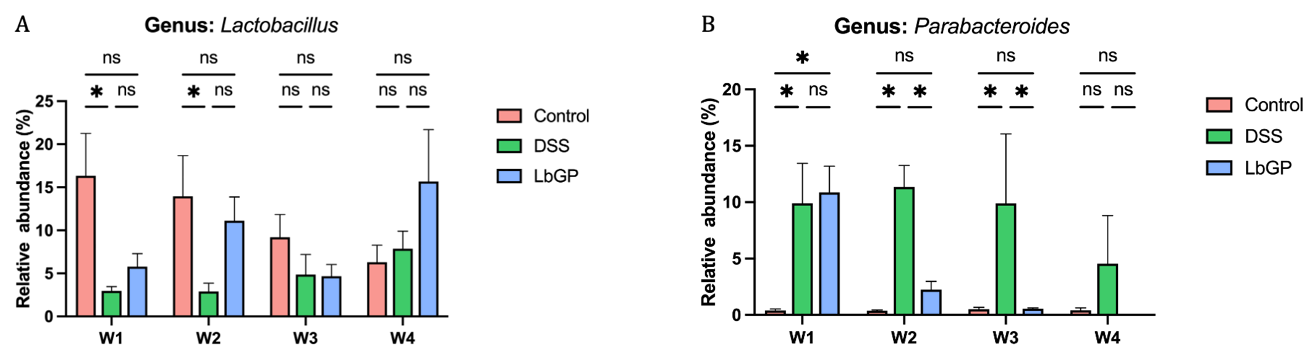


**Supplementary Figure S4.** The relative abundance of *Lactobacillus* (A) and *Parabacteroides* (B). (n=5, **P*<0.05).


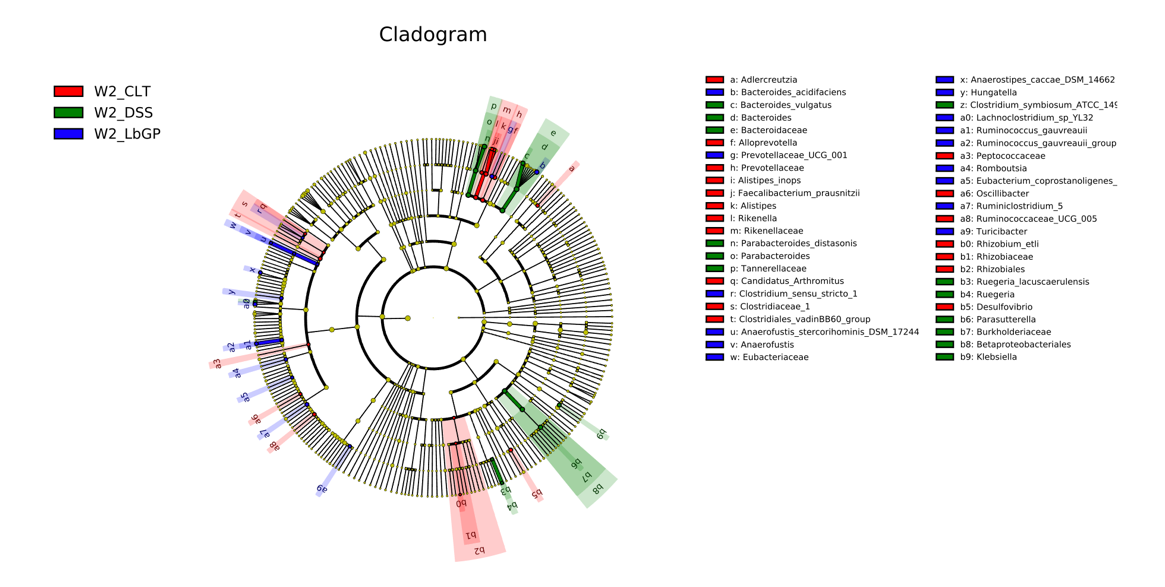


**Supplementary Figure S5.** The cladogram of LEfSe analysis among the Control group, DSS group, and LbGP group at W2.


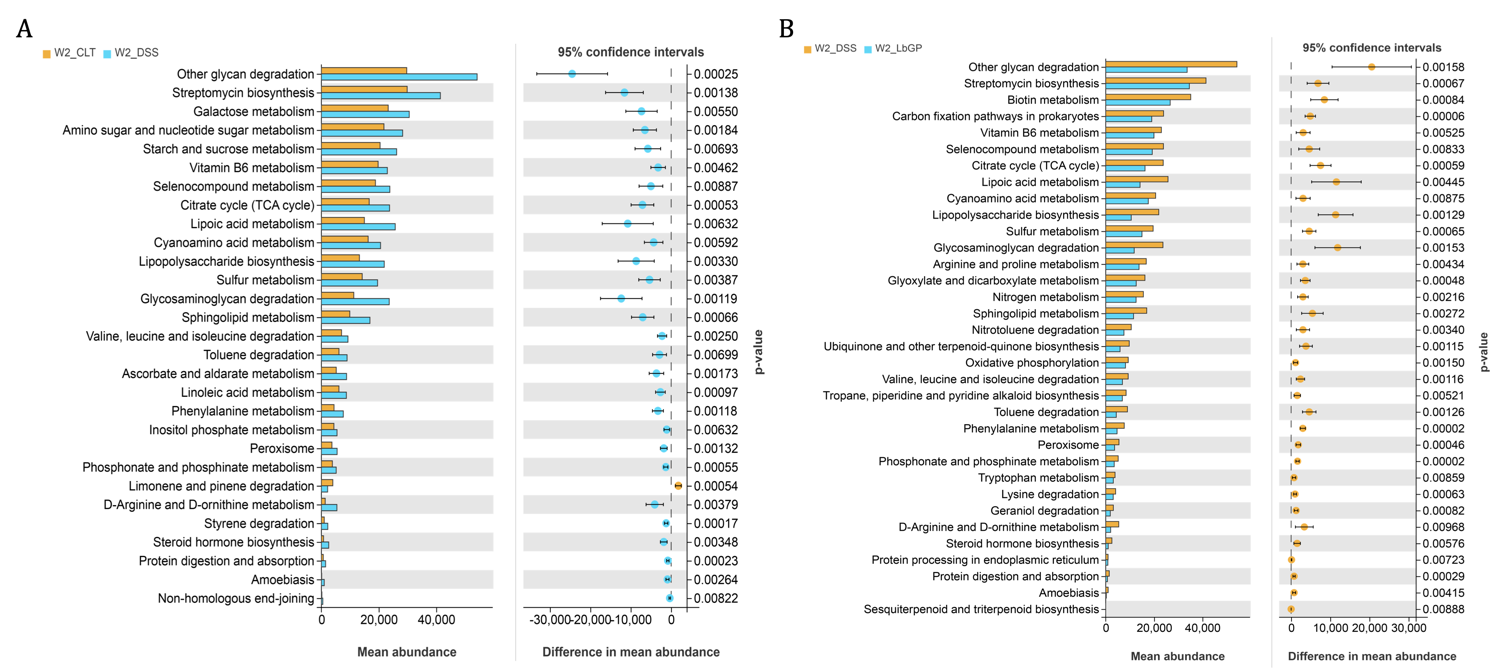


**Supplementary Figure S6.** KEGG Orthology enrichment between groups. (A) Differential KEGG pathway of level 3 between the control group and DSS group at W2 under Welch’s T test. (B) Differential KEGG pathway of level 3 between the DSS group and LbGP group at W2 under Welch’s T test (n=5).

# Supplementary Table

Supplementary Table S1. Primer sequences

| primer | Primer sequences |
| --- | --- |
| GAPDH | F: 5’- TGGCCTTCCGTGTTCCTAC-3’ |
|  | R: 5’- GAGTTGCTGTTGAAGTCGCA-3’ |
| IL-1β | F: 5’- TTCAGGCAGGCAGTATCACTC-3’ |
|  | R: 5’- GAAGGTCCACGGGAAAGACAC-3’ |
| IL-6 | F: 5’- CCCCAATTTCCAATGCTCTCC -3’ |
|  | R: 5’- CGCACTAGGTTTGCCGAGTA -3’ |
| IL-10 | F: 5’- CGGGAAGACAATAACTGCACCC -3’ |
|  | R: 5’- CGGTTAGCAGTATGTTGTCCAGC -3’ |
